# Silver Nanoparticles Seed Priming for Sustainable Enhancement of Durum Wheat Growth, Yield, and Nutrient Enrichment

**DOI:** 10.1049/nbt2/6152486

**Published:** 2025-02-19

**Authors:** Yasser Al Salama, Ibrahim Alghoraibi, Raghad Zein, Mohammad Alsouse

**Affiliations:** ^1^Department of Soil and Land Reclamation, Faculty of Agriculture, Al-Furat University, Deir ez-Zor, Syria; ^2^Department of Physics, Faculty of Science, Damascus University, Damascus, Syria

**Keywords:** crop, morphological indicators, nano silver, sustainable food security

## Abstract

Achieving food security stands as a primary challenge confronting global societies today. This necessitates the development of effective strategies to increase crop productivity and enhance their specifications, aiming to meet the growing market demands sustainably and efficiently. This research was conducted over two agricultural seasons and emphasizes the ability of silver nanoparticles (AgNPs) to promote the growth and productivity of durum wheat (variety Sham 7) cultivated under the conservative conditions of Deir ez-Zor. The wheat seeds were soaked before planting with a colloidal suspension of AgNPs, prepared through an eco-friendly method utilizing an aqueous extract of *Eucalyptus camaldulensis* leaves. The best plant morphological indicators (plant height, chlorophyl content, number of branches, and number of spikes) were observed when colloidal AgNPs were used as a soaking solution compared with silver nitrate (AgNO_3_)and deionized (DI) water as a control. The highest productivity parameters (grain yield, straw yield, and 1000-grain weight) were obtained when seeds were soaked in 40 ppm of AgNPs. Furthermore, the results revealed an increase in the nutrient content of grain (nitrogen, phosphorus, and potassium). This study offers valuable insights into the prospective use of AgNPs for significant improvement in wheat cultivation, increasing productivity, and improving crop quality. As a contribution to facing future challenges in the field of agriculture and ensuring sustainable food security.

## 1. Introduction

Throughout history, agriculture has served as the cornerstone of economies, contributing significantly to the formation of society. In today's world, ensuring food security is of utmost importance. Wheat cultivation is a fundamental pillar for addressing this challenge. This vital cereal crop plays a unique role, feeding over 2 billion people worldwide in accounting for nearly 40% of the global population [1]. Considering the limited arable land, maximizing wheat production per unit area becomes imperative. However, traditional farming methods using regular fertilizers and pesticides harm the environment in the long run, which underscores the critical need for innovative approaches.

Nanotechnology holds great promise for improving crop production [2, 3]. Nanomaterials hold promise for enhancing plant germination and crop yield, enhancing plant resistance to both environmental and biological stress factors, optimizing nutrient utilization, and promoting overall plant growth [4–6]. Importantly, these effects are achieved with a lower environmental impact than conventional methods using bulk materials. Their effectiveness is largely affected by the type of nanomaterials, their concentration, exposure duration, and the specific plant species [7, 8]. Silver nanoparticles (AgNPs) produced using green methods exhibit distinctive characteristics attributed to their nanoscale dimensions, including a significantly enlarged surface area, reactivity, and stability. Incorporating these environmentally friendly nanoparticles into production methods has been proven to enhance plant stress tolerance [9–11], shoot multiplication [12], and overall yield of plant-based products [13–15]. The use of AgNPs in agriculture offers significant benefits, including enhanced plant growth, improved resistance to pathogens, and reduced reliance on chemical fertilizers and pesticides. However, these benefits are accompanied by potential environmental risks, such as soil microbial disruption, nanoparticle accumulation, and toxicity to nontarget organisms as well as cost implications. While AgNPs may offer long-term savings by improving crop yields, their initial production costs and potential environmental remediation expenses must be considered. Seed priming, which is a brief pregermination soaking, has positive effects on improving germination and subsequent plant traits. This involves soaking seeds in distilled water or various nutrient solutions. AgNP seed priming is a highly effective method to harness the benefits of AgNPs while mitigating their potential negative effects. This approach involves direct application to seeds, ensuring efficient absorption and significantly reducing the amount of AgNPs introduced into the soil. Consequently, this minimizes their environmental impact on soil and the surrounding ecosystem, making it a sustainable technique in agricultural practices. Pregermination soaking with AgNPs plays an important role in various physiological aspects of wheat plants [16, 17]. It enhances grain germination [18] and seedling growth [19, 20], influence root number [21], increase root length, dry weight [22, 23], and stimulate protein synthesis [24].

This study investigates the effect of AgNPs in enhancing the morphological features of durum wheat (variety Sham7) grown under the conditions of Deir ez-Zor Governorate in Syria. A colloidal suspension of green-synthesized AgNPs was used. Morphological indicators such as plant height, branch number, spike number, and chlorophyl content were studied. In addition, straw yield, grain yield, 1000-grain weight, and nutritional elements such as nitrogen, potassium, and phosphorous content were estimated.

## 2. Materials and Methods

### 2.1. Experimental Site

This study was carried out at the Agricultural Scientific Research Centre at Saalou Research Station, located 30 km east of Deir ez-Zor city (35°19′ N 40°08′ E, 203 ma.s.l.). It was conducted over two agricultural seasons 2021–2022 and 2022–2023. The land was plowed and divided into experimental plots of 2 m^2^ each. The fundamental physical and chemical characteristics of the soil are illustrated in Table 1.

### 2.2. Synthesis of AgNPs

AgNPs were prepared at Nano Lab in the Physics Department at Damascus University as mentioned in the previous work of Zein et al. [25].

In brief, a solution of silver nitrate (AgNO_3_) was combined with an aqueous extract of *Eucalyptus camaldulensis* leaves, resulting in a noticeable color change. This alteration indicates the formation of AgNPs. The mixture was subsequently placed in a dark environment for 24 hr, ensuring that the reaction reached its completion. Following this, the nanoparticles were separated from the mixture using centrifugation for 5 min. The resulting residue was subjected to four rounds of washing with deionized (DI) water followed by ethanol, with centrifugation employed as the method of removal for both silver ions and any residual organic materials.

A colloidal suspension of AgNPs was formulated by adding DI water to purified AgNPs. Following comprehensive dispersion through an ultrasonic bath, the suspension was stored for subsequent use.

### 2.3. Experimental Design

**Figure 1:**
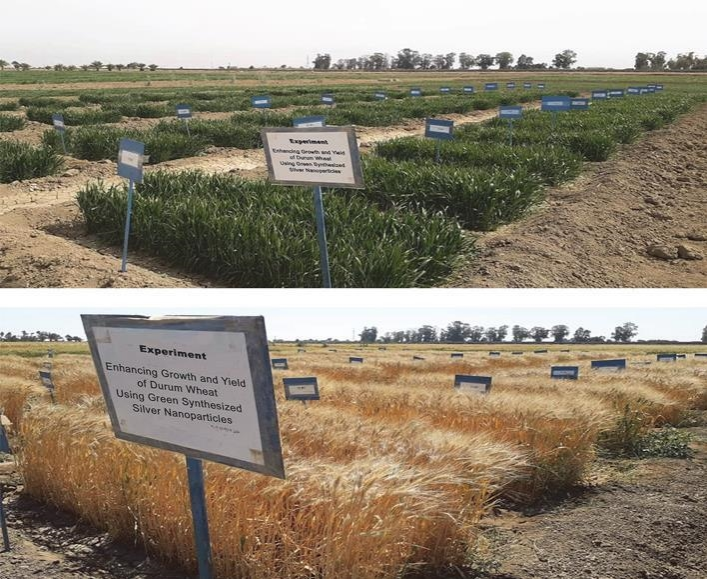
The experimental design of durum wheat (*Triticum durum L*., variety Sham 7).

Durum wheat grains (*Triticum durum L*., variety Sham 7) were divided into three groups. The first and second groups were soaked in AgNO_3_ solution and AgNPs, respectively, at concentrations of 10, 20, 40, and 80 ppm. The third group was soaked in distilled water as a control sample. The soaking process lasted for 2 hr before planting. Subsequently, the grains were sown at a rate of 200 kg/ha, on four lines with a spacing of 25 cm between each line (Figure 1). Mineral fertilizers (NPK) were applied based on the agricultural recommendations provided by the Ministry of Agriculture and Land Reclamation for irrigated wheat cultivation. After reaching full growth, 10 plants were randomly selected from each experimental plot for morphological measurements. The height of the plant was measured at each stage: tillering, heading, and harvesting using a ruler extended from the base to the tip. The average number of branches and spikes was counted. The total content of chlorophyl was measured using a field chlorophyl meter (SPAD) at the beginning of the heading stage. The grain yield, straw yield, and average weight of 1000 grains were estimated for each experimental plot and concentration, with four replicates. Additionally, the grain content of nitrogen, phosphorus, and potassium was determined according to Van Schouwenberg and Walinge [26], Page, Miller, and Keeney [27], and Rashid [28].

The field experiment was conducted following a factorial experiment design with two factors (type and concentration of soaking solution) and four replicates for each treatment.

### 2.4. Statistical Analysis

The collected data underwent statistical analysis using the MSTATC software. The least significant difference (LSD) values at a significance level of 0.05 were used to compare the treatment means.

## 3. Results

This research explored how the yield and growth of durum wheat are influenced by AgNPs synthesized using a green method as mentioned previously by Zein et al. [25]. An aqueous *Eucalyptus Camaldulensis* leaves extract was used as reducing agent to prepare AgNPs. The formation of AgNPs was confirmed using UV-visible spectroscopy, with a surface plasmon peak at 449 nm. The particle size was determined by scanning electron microscopy (SEM), dynamic light scattering (DLS), and nanoparticle tracking analysis (NTA), with average sizes of 12, 10, and 23 nm, respectively. Additionally, energy dispersive X-ray spectroscopy (EDS) analysis confirmed that silver was the main element. Zeta potential measurements of −23 mV indicated good stability of the nanosilver formulation, owing to the repulsion between nanoparticles, which reduces agglomeration.

There was a significant (*p* < 0.05) effect of AgNPs on morphological indicators, productivity parameters and nutrient content of wheat grains (Tables 2–4).

The data presented in Table 2 reveal the morphological attributes of wheat plants (plant height at different phenological stages, chlorophyl content, number of branches, and number of spikes) at different concentrations (10, 20, 40, and 80 ppm) of soaking materials (AgNO_3_ and AgNPs) compared with the control (DI water). Soaking seeds in the control resulted in plant height of 27.90, 70.13, and 84.30 cm at various phenological stages (tillering, heading, and harvest), respectively.

The highest heights of wheat plant were observed after soaking seeds with 10 ppm concentration of colloidal AgNPs. The heights were 34.95, 85.92, and 96.58 cm at different stages (tillering, heading, and harvest), respectively. The heights decreased by increasing the concentrations of AgNPs to 20, 40, and 80 ppm.

The same result was obtained with AgNO_3_. The plant heights decreased when the concentrations more than 20 ppm were used (40 and 80 ppm). The tallest plants were showed at 20 ppm (31.88, 74.68, and 87.88 cm). The results revealed a difference in chlorophyl content depending on the soaking solution and concentrations (10, 20, 40, and 80 ppm). The highest value of chlorophyl content was obtained with 10 ppm of AgNPs by 35.87. The number of branches and spikes also differs according to the soaking material. Soaking seeds in DI water and AgNO_3_ had no significant differences in the number of branches and spikes per plant. Using AgNPs led to a noticeable increase in the number of branches and spikes compared to DI water. The maximum number of branches and spikes were observed at 40 ppm of AgNPs with 5.78 and 6.71, respectively.

The impact of soaking wheat seeds before planting with different concentrations (10, 20, 40, and 80 ppm) of AgNPs, AgNO_3_, and DI water on some productivity indicators (straw yield, grain yield, and 1000-grain weight) is illustrated in Table 3. The results clearly showed a significant increase in wheat plant productivity when seeds were soaked in the colloidal AgNPs. The results indicated that soaking seeds in colloidal AgNPs at a concentration of 40 ppm yielded the best results of productivity (10.453 tons/ha, 9.746 tons/ha, and 50.89 g) compared to AgNO_3_ (8.764 tons/ha, 8.024 tons/ha, and 50.34 g) and distilled water (7.43 tons/ha, 6.67 tons/ha, and 48.67 g) for straw yield, grain yield, and 1000-grain weight, respectively. On the other hand, the productivity indicators of wheat plants decreased at 80 ppm of AgNPs but still higher than the control.

**Figure 2:**
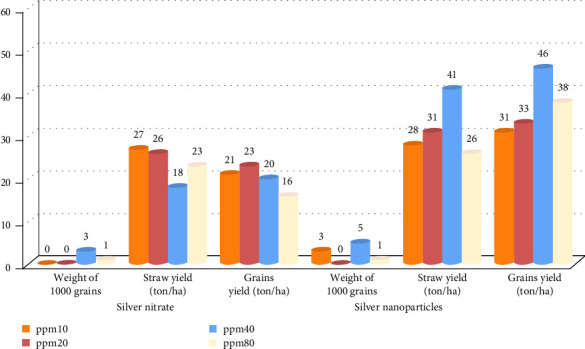
The percentage increase in productivity traits of wheat plants (straw yield, grain yield, and 1000-grain weight) for both soaking materials (silver nitrate (AgNO_3_) and silver nanoparticles (AgNPs)) at different concentrations (10, 20, 40, and 80 ppm) compared to the control (deionized (DI) water).

The percentage increase in productivity traits of wheat plants due to soaking the seeds in both AgNO_3_ and AgNPs is shown in Figure 2. It indicated a noticeable increase in straw yield, grain yield, and 1000-grain weight when the seeds are soaked in the colloidal AgNPs as opposed to AgNO_3_. The findings suggest that immersing the seeds in the AgNPs suspension at a concentration of 40 ppm resulted in the highest percentage rise in straw yield (41%), grain yield (46%), and 1000-grain weight (5%).

The impact of preplanting soaking of wheat seeds with different concentrations of green synthesized AgNPs and AgNO_3_ (10, 20, 40, and 80 ppm) on the nutrient composition of grains, particularly nitrogen, phosphorus, and potassium is presented in Table 4. Immersing the seeds in a solution of AgNO_3_ and AgNPs significantly led to an increase in the nutrient content. The higher content of nutrients was observed at a concentration of 40 ppm. The content of nitrogen, phosphorus, and potassium was 3.298%, 0.5432%, 0.5723% and 3.425%, 0.8745%, 0.5967% for soaking in AgNO_3_ and AgNPs, respectively. It is higher than the amount of nutrients when soaking with distilled water (2.940%, 0.3876%, and 0.5243%). The nutrient content decreases when the concentration of soaking material increases.

**Figure 3:**
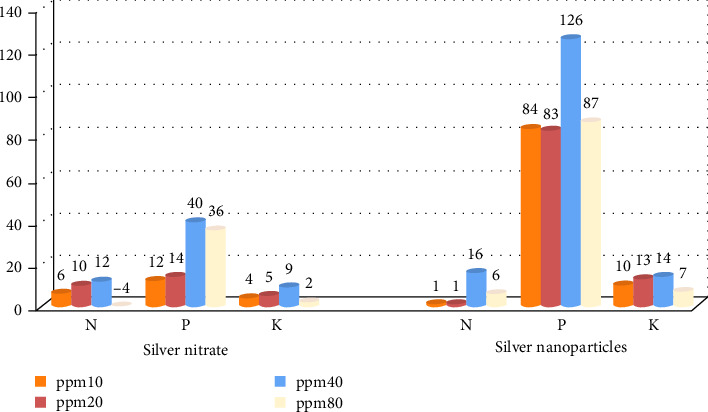
Percentage enhancement in nitrogen (N), phosphorus (P), and potassium (K) concentrations in wheat grains for both soaking materials (silver nitrate (AgNO_3_) and silver nanoprticles (AgNPs) at different concentrations (10, 20, 40, and 80 ppm) compared with the control (deionized (DI) water).

The percentage rise in nutrient concentrations (nitrogen, phosphorus, and potassium) in wheat grains following the soaking of seeds in different concentrations (10, 20, 40, and 80 ppm) of both AgNO_3_ and AgNPs suspension compared to the control (distilled water) is showed in Figure 3. A significant increase in phosphorus levels was observed due to seed soaking in the colloidal of AgNPs and AgNO_3_, with the increase exceeding 80% due to soaking with AgNPs at different concentrations. However, the increases in both nitrogen and potassium did not exceed 15% in either case, regardless of the applied concentrations. It was found that soaking the seeds in AgNPs at a concentration of 40 ppm yielded the most substantial increase in the percentages of the examined elements. Specifically, N, P, and K levels increased by 16%, 126%, and 14%, respectively.

## 4. Discussion

It demonstrated statistically significant differences in the results of the field experiment for the studied wheat plant traits. Soaking the wheat seeds in green synthesized AgNPs or AgNO_3_ at concentrations of (10, 20, 40, and 80 ppm) resulted in a notable enhancement of the examined morphological characteristics. The highest plant heights at different phenological stages (tillering, heading, and harvest) were observed at 10 ppm concentration of AgNPs compared with AgNO_3_ and the control. The results were aligned with the discoveries of Pallavi et al. [22], which demonstrated that soaking seeds with 50 ppm AgNPs led to an increase in wheat plant stem length. Additionally, Iqbal et al. [29] showed that AgNPs improved several morphological and phenological indicators of wheat seedlings (three-leaf stage) at concentrations of 50 and 75 ppm. The heights decreased by increasing the concentration of AgNPs to 20, 40, and 80 ppm. This negative effects on plant growth because of the toxicity of AgNPs, which is concentration and size-dependent [30]. Its accumulation in plants affects their defence mechanisms, alters various biochemical activities, and leads to changes in their appearance and health. These changes can negatively impact processes like photosynthesis and cause irregular growth patterns and symptoms in the plants [31].

Soaking the wheat grains in AgNPs which are synthesized using *E. camaldulensis* leaves extract increased the chlorophyl content and the number of branches and spikes per plant. Similar results were observed by Farghaly and Nafady [32] and Ahmed et al. [33] when wheat grains were treated with AgNPs, the seedling length and chlorophyl content were significantly increased relative to the control.

AgNPs revealed a significant effect on the productivity traits of wheat plants. Maximum yields were recorded with AgNPs at 40 ppm. Applying a higher concentration (80 ppm) of AgNPs inhabited the growth and negatively affected the straw and grain yield and the weight of 1000-grain. This is in line with the results obtained by Sadak [34], who reported that the treatments with lower concentrations of AgNPs significantly increase photosynthetic pigment contents in fenugreek plants, which in turn promote the photosynthesis process and increase the yield and growth of plants. Verma, Patel, and Kushwah [35] reported that AgNPs increased the root and shoot length, plant growth, photosynthetic pigments, and yield quantity of treated *Phaseolus vulgaris L*. plant with 60 mg/L concentration. While higher concentrations of AgNPs revealed a toxic effect on the morphological and phytochemical parameters of *P. vulgaris L*.

A noticeable change in the nutrient content of nitrogen, phosphorus, and potassium was observed when seeds were soaked in a colloidal suspension of AgNPs and AgNO_3_ compared with the control treatment (DI water) and that aligns with what has been mentioned in several previous studies. Guzmán-Báez et al. [36] found that AgNPs increased the concentrations of N, P, and K in tomato (*Solanum lycopersicum*) leaves. Razzaq et al. [37] observed that the quality of NPK uptake and nutrient efficiency was improved in wheat after using chemically synthesized AgNPs. Yuvaraj and Sevathapandian Subramanian [38] showed that AgNPs as nano fertilizers effectively prevent the loss of nutrients and help the plant to enhance nutrient absorption from the soil.

## 5. Conclusion

AgNPs play a crucial role in enhancing agricultural productivity, particularly at low concentrations where they significantly improve chlorophyl content plant heights, yield, and phosphorus content. However, higher concentrations may have negative effects on morphological attributes and nutrient content. Despite this, the utilization of green synthesized AgNPs presents promising prospects for improving plant growth, especially in drought-prone environments, thus, contributing to future agricultural challenges and sustainable food security.

## Figures and Tables

**Table 1:** Soil physical and chemical properties of the experimental sites.

Available minerals			CaCO_3_ (%)	OM (%)	E.C. (ds/m)	pH
K (ppm)	P (ppm)	N (%)				
406	7.5	0.16	2.25	1.07	2.32	7.8

**Bulk density (g/cm^3^)**	**Apparent density (g/cm^3^)**	**Total porosity (%)**	**Soil texture**	**Particle size distribution (%)**		
				**Clay**	**Silt**	**Sand**

2.45	1.45	40.81	Clay	46	22	32

**Table 2:** Morphological attributes of wheat plants (plant height at different phenological stages, chlorophyl content, number of branches, and number of spikes) for both soaking materials (silver nitrate (AgNO_3_) and silver nanoparticles (AgNPs)) at different concentrations (10, 20, 40, 80 ppm) and control (deionized (DI) water).

Material type	Concentration (ppm)	Plant height (cm)			Chlorophyl SPAD	Number of branches/plant	Number of spikes/plant
		Tillering	Heading	Harvest			
Control	0	27.90^f^	70.13^ef^	84.30^e^	33.59^e^	4.48^bcd^	5.13^ef^

AgNO_3_	10	30.00^e^	74.57^de^	84.65^de^	35.12^abc^	4.51^bc^	5.24^ef^
	20	31.88^e^	74.68^de^	87.88^cd^	35.73^abcd^	4.57^bcd^	5.43^cdef^
	40	31.63^de^	74.28^d^	86.02^de^	35.67^abcd^	4.95^bc^	5.23^def^
	80	30.74^e^	73.75^de^	85.28^e^	35.80^bcd^	4.40^bcd^	5.63^cde^

AgNPs	10	34.95^a^	85.92^de^	96.58^a^	35.87^a^	5.76^a^	6.65^ab^
	20	34.41^ab^	84.74^a^	94.09^ab^	35.69^ab^	5.68^a^	6.43^abc^
	40	33.95^bcd^	83.98^ab^	91.45^bc^	34.96^abcd^	5.78^a^	6.71^a^
	80	33.15^bc^	81.45^bc^	90.40^bc^	34.88^abcd^	5.33^ab^	6.03^bcd^

LSD_0.05_	—	1.76	3.97	3.22	0.78	0.657	0.674

*Note:* Values are the means of four replicates. Different alphabets differ significantly from each other at *p* < 0.05.

Abbreviation: LSD, least significant difference.

**Table 3:** Productivity indicators of wheat plants (straw yield, grain yield, and 1000-grain weight) for both soaking materials (silver nitrate (AgNO_3_) and silver nanoparticles (AgNPs)) at different concentrations (10, 20, 40, and 80 ppm), and control (deionized (DI) water).

Material type	Concentration (ppm)	Grain yield (tons/ha)	Straw yield (tons/ha)	1000 grain weight (g)
Control	0	6.670^f^	7.43^h^	48.67^abc^

AgNO_3_	10	8.092^cd^	9.423^c^	48.45^abc^
	20	8.233^c^	9.363^def^	48.68^abc^
	40	8.024^cd^	8.764^def^	50.34^ab^
	80	7.734^cde^	9.132^cde^	48.94^ab^

AgNPs	10	8.755^b^	9.534^b^	50.32^ab^
	20	8.843^b^	9.724^b^	48.76^abc^
	40	9.746^a^	10.453^a^	50.89^a^
	80	9.173^b^	9.354^b^	49.23^ab^

LSD_0.05_	—	0.26	0.42	2.98

*Note:* Values are the means of four replicates. Different alphabets differ significantly from each other at *p* < 0.05.

Abbreviation: LSD, least significant difference.

**Table 4:** The nutritional content (nitrogen (N), phosphorus (P), and potassium (K)) of wheat grains for both soaking materials (silver nitrate (AgNO_3_) and silver nanoparticles (AgNPs)) at different concentrations (10, 20, 40, and 80 ppm) and control (deionized (DI) water).

Material type	Concentration (ppm)	N (%)	P (%)	K (%)
Control	0	2.940^g^	0.3876^f^	0.5243^bc^

AgNO_3_	10	3.124^e^	0.4356^e^	0.5467^bc^
	20	3.245^d^	0.4430^d^	0.5523^ab^
	40	3.298^c^	0.5432^c^	0.5723^ab^
	80	2.834^c^	0.5278^c^	0.5324^ab^

AgNPs	10	2.965^b^	0.7123^b^	0.5764^ab^
	20	2.981^a^	0.7098^b^	0.5923^a^
	40	3.425^a^	0.8745^a^	0.5967^a^
	80	3.123^a^	0.7267^b^	0.5634^a^

LSD_0.05_	—	0.123	0.035	0.0152

*Note:* Values are the means of four replicates. Different alphabets differ significantly from each other at *p* < 0.05.

Abbreviation: LSD, least significant difference.

## Data Availability

The data that support the findings of this study are available upon reasonable request.
